# The effects of graded caloric restriction: XII. Comparison of mouse to human impact on cellular senescence in the colon

**DOI:** 10.1111/acel.12746

**Published:** 2018-03-25

**Authors:** Luigi Fontana, Sharon E. Mitchell, Boshi Wang, Valeria Tosti, Thijmen van Vliet, Nicola Veronese, Beatrice Bertozzi, Dayna S. Early, Parcival Maissan, John R. Speakman, Marco Demaria

**Affiliations:** ^1^ Division of Geriatrics and Nutritional Sciences and Center for Human Nutrition Washington University School of Medicine St. Louis MO USA; ^2^ Department of Clinical and Experimental Sciences Brescia University Brescia Italy; ^3^ Institute of Biological and Environmental Sciences University of Aberdeen Aberdeen UK; ^4^ European Research Institute for the Biology of Aging University Medical Center Groningen University of Groningen Groningen The Netherlands; ^5^ State Key Laboratory of Molecular Developmental Biology Institute of Genetics and Developmental Biology Chinese Academy of Sciences Beijing China

**Keywords:** ageing, aging, caloric restriction, cellular senescence, SASP

## Abstract

Calorie restriction (CR) is an effective strategy to delay the onset and progression of aging phenotypes in a variety of organisms. Several molecular players are involved in the anti‐aging effects of CR, but mechanisms of regulation are poorly understood. Cellular senescence—a cellular state of irreversible growth arrest—is considered a basic mechanism of aging. Senescent cells accumulate with age and promote a number of age‐related pathologies. Whether environmental conditions such as diet affect the accumulation of cellular senescence with age is still unclear. Here, we show that a number of classical transcriptomic markers of senescent cells are reduced in adult but relatively young mice under CR. Moreover, we demonstrate that such senescence markers are not induced in the colon of middle‐age human volunteers under CR in comparison with age‐matched volunteers consuming normal Western diets. Our data support the idea that the improvement in health span observed in different organisms under CR might be partly due to a reduction in the number of senescent cells.

## INTRODUCTION

Human lifespan and health span have risen significantly in recent decades (Vaupel, 2010). Yet, aging is a progressive and generalized deterioration of the functional capacities of an organism which strongly contributes to tissue failure. Accordingly, age is one of the largest single risk factors for developing diseases, from neurodegeneration to cancer. The effects of aging are largely influenced by genetic and environmental conditions. While genetic manipulations of model organisms have set important milestones for the understanding of the aging process, calorie restriction (CR) is a well‐established nongenetic approach able to improve health span and lifespan in different organisms (Finkel, 2015). However, the precise mechanisms by which CR improves health are not fully understood (Speakman & Mitchell, 2011; Fontana & Partridge, 2015).

More than 50 years ago, Hayflick and Moorhead found that human diploid cell strains have a definite lifespan due to the activation of a state of growth arrest after extensive serial passages in culture. They described this phenomenon as “cellular senescence” and postulated its importance during aging (Hayflick & Moorhead, 1961). Subsequent studies demonstrated that senescent cells gradually accumulate with increasing age in various organisms (Loaiza & Demaria, 2016). During aging, senescent cells impair cellular turnover and tissue regeneration due to their inability to proliferate, and stimulate a pro‐disease environment by the chronic secretion of various pro‐inflammatory and tissue‐remodeling factors, a phenotype called Senescence‐Associated Secretory Phenotype (SASP; Loaiza & Demaria, 2016).

Genetic and pharmacological elimination of senescent cells is sufficient to improve health span (Soto‐Gamez & Demaria, 2017). Interestingly, a previous report suggested that CR prevented accumulation of senescent cells in the mouse liver and intestine (Wang et al., 2010). To further explore the potential reduction in senescent cells upon short‐term CR, and whether this phenomenon might potentially happen in humans, we analyze various classical transcriptomic markers for senescence and SASP in short‐term CR interventions in the mouse and human colon mucosa specimens.

**Figure 1:**
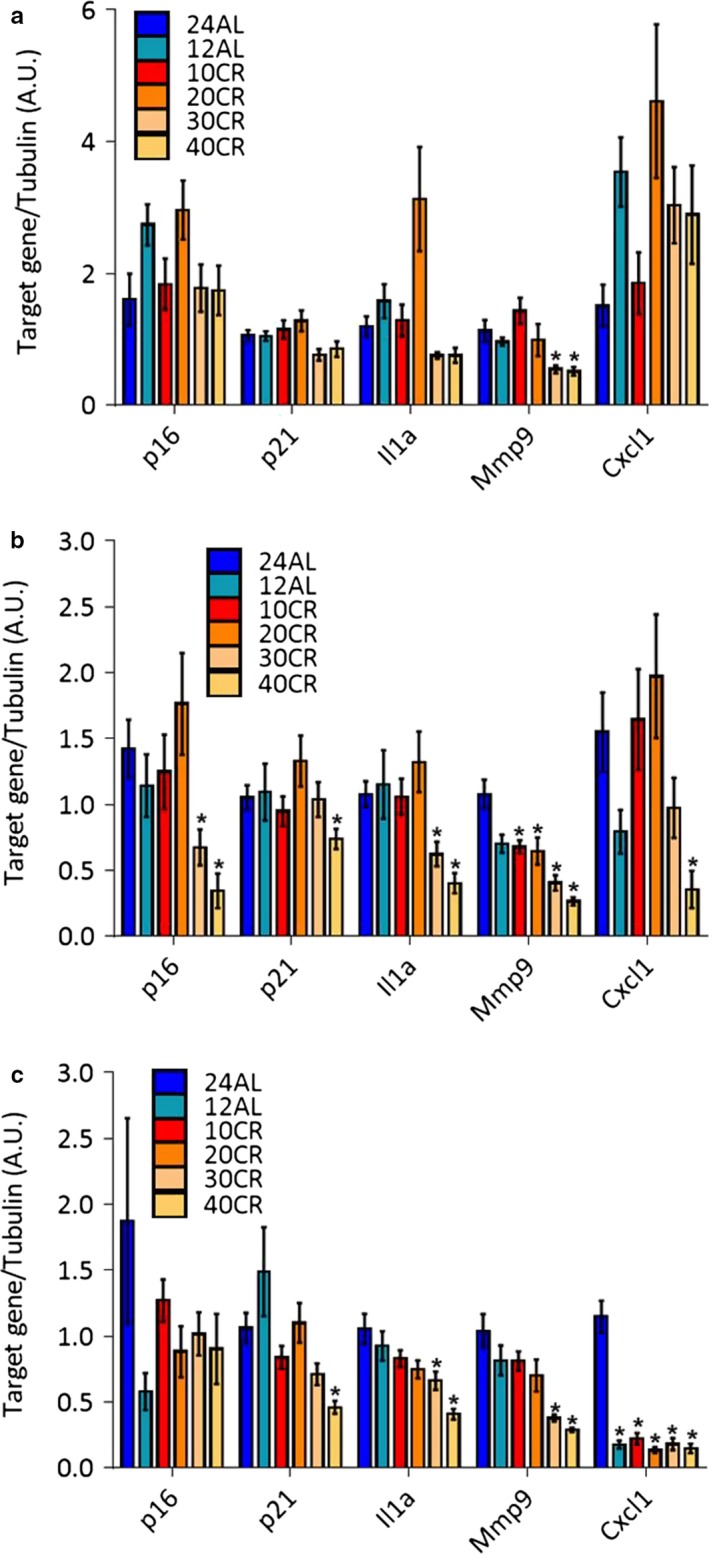
Expression of senescence‐associated genes in control or calorie restricted (CR) mouse colon. RNA was extracted from the proximal (a), medial (b), or distal (c) colon of mice with 24 or 12 hr ad libitum access to food (24AL and 12AL, respectively) or mice under 10%, 20%, 30%, or 40% calorie restriction (10CR, 20CR, 30CR, and 40CR, respectively). mRNA encoding p16, p21, Il1a, Mmp9, and Cxcl1 were quantified by qRT–PCR. mRNA encoding tubulin was used as internal control. *N* = 12–18. *p < .05

Male mice were aged 20 weeks when they entered four levels of CR for 12 weeks: 10%, 20%, 30%, and 40% restriction from baseline food intake (Mitchell et al., 2015). Two control groups, 12‐ and 24‐hr ad libitum access to food (12AL and 24AL, respectively), were used, and statistical analysis was calculated using 24AL as reference. The colon of these mice was divided into three regions: proximal, medial, and distal. In the proximal colon, the expression levels of two classical markers of senescence‐associated growth arrest, the cyclin‐dependent kinase inhibitors p16 and p21, did not change significantly among groups (Figure 1a). Selected markers for the SASP (Il1a, Mmp9, and Cxcl1) also did not significantly change with the exception of mmp9 which was downregulated at 30% and 40% CR regimens (Figure 1a). In the medial colon, while there were no differences among the two controls and the lowest CR interventions (10%–20%), p16, p21, Il1a, Mmp9, and Cxcl1 were all downregulated at higher CR regimens, with stronger statistical significance in the CR 40% group (Figure 1b). A similar trend was present in the distal colon with the exception of p16, which lower level compared to AL24 did not reach statistical significance in any group (Figure 1c). These data suggest that short‐term CR at higher levels can prevent or decrease the accumulation of senescent cells in the mouse colon, even in adult but relatively young animals on short‐term restriction.

**Figure 2:**
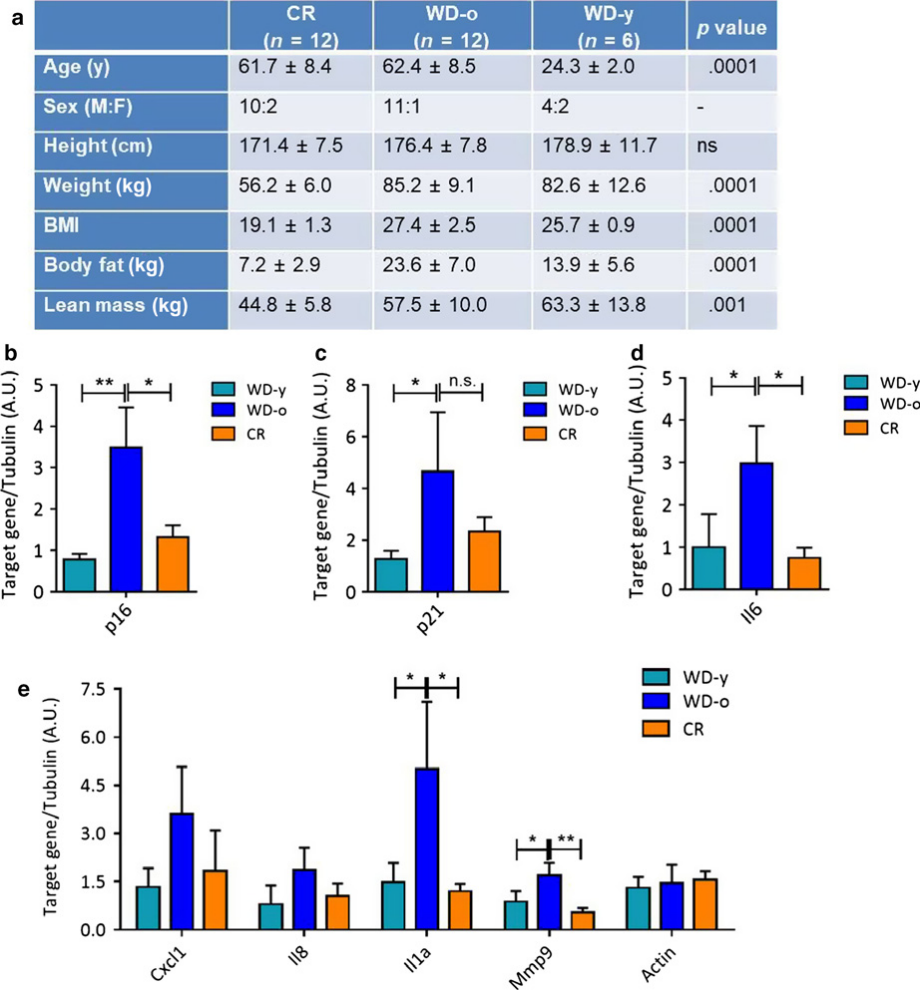
Expression of senescence‐associated genes in control or calorie restricted (CR) human colon. RNA was extracted from the sigmoid portion of the colon of human volunteers. The groups were as follows: CR, volunteers of average age 61.7 ± 8.4 under <15% calorie restriction; WD‐o, volunteers of average age 62.4 ± 8.5 on normal Western diet; WD‐y, volunteers of average age 24.3 ± 2.0 on normal Western diet. A summary is prided in a. mRNA encoding p16 (b), p21 (c), Il6 (d) and other SASP factors Cxcl1, IL‐8, Il1a, and Mmp9 (e) were quantified by qRT–PCR. mRNA encoding tubulin was used as internal control. In E, dotted line represents the baseline value of WD‐y samples. *N* = 6, WD‐y; *N* = 12, CR and WD‐o. *p < .05; **<.01

We then sought to determine whether CR modifies the expression levels of senescence and SASP markers in the human sigmoidal colon mucosa (Data S1). To this end, we recruited and studied 12 middle‐aged (61.7 ± 8.4 years), weight‐stable very lean (BMI = 19.1 ± 1.3 kg/m^2^) members of the Calorie Restriction Society who have been practicing ~30% CR with adequate nutrition (at least 100% of RDI for each nutrient) for an average of 10.1 years (Most, Tosti, Redman & Fontana, 2017; Yang et al., 2016) and a control group of 12 nonobese (BMI = 27.4 ± 2.5 kg/m^2^) age‐matched sedentary controls eating a typical Western diet (WD‐o; Figure 2a). Furthermore, we compared the CR and WD‐o groups with younger (24.3 ± 2.0 years, range 21–27 years) nonobese (BMI = 25.7 ± 0.9 kg/m^2^) humans (WD‐y). All the genes measured were expressed at higher level in WD‐o than in WD‐y volunteers (Figure 2b–e). Levels of p16 were significantly lower in the CR compared to WD‐o volunteers (Figure 2b). Levels of p21 followed the trend observed in p16, but did not reach statistical significance (Figure 2c). In accordance with a previous study, we observed significantly lower level of the pro‐inflammatory cytokine IL‐6 in the CR colon mucosa (Figure 2d; You, Sonntag, Leng & Carter, 2007). The other SASP factors analyzed Cxcl1, Il8, Il1a, and Mmp9 followed similar trends, but only the latter two reached statistical significance (Figure 2e). Tubulin was used as internal reference gene, and mRNA levels of another housekeeping gene, actin, were also unchanged among groups (Figure 2e). These data suggest that CR could potentially prevent the accumulation of age‐associated senescent cells in the colon mucosa of human beings, and the reduction in senescence might explain the much lower levels of inflammation observed in CR individuals (Meydani et al., 2016).

The hypothesis of cellular senescence as a basic mechanism of aging is increasingly supported by experimental evidence (Childs et al., 2017). Senescent cells are visible during aging and at sites of age‐related pathologies in both human and mice (Loaiza & Demaria, 2016; Childs et al., 2017). The use of genetic models showed that elimination of senescent cells can reduce age‐related pathologies and improve health span and lifespan (Demaria et al., 2017; Jeon et al., 2017; Baker et al., 2016). Senolytics are currently under development, but intrinsic toxicities and nonspecificity of the current antisenescence drugs are hurdles for long‐term treatments to interfere with aging in humans (Soto‐Gamez & Demaria, 2017).

Calorie restriction is a potent intervention for delaying aging and age‐related pathologies, but the factors determining these effects are largely unknown (Fontana & Partridge, 2015). The reduced expression of markers of senescence in both humans and mice is an intriguing mechanism that could further explain the potential beneficial effects of CR. This study re‐enforces the importance of dietary interventions for senescence induction or prevention. Indeed, CR was previously shown to reduce senescence in the mouse liver and intestine (Wang et al., 2010), and high‐fat diet was recently implicated in promoting accelerated senescence with detrimental effects in mice (Schafer et al., 2016). Of course, more studies are warranted to understand how lowering calorie intake reduces senescence burden, and whether the reduction in senescence is sufficient to directly lower the levels of various tissue‐remodeling factors and interleukins, which could be affected by several other variables independently perturbed by the presence of senescent cells. Specifically for the colon, it will be of interest to investigate the cell types that undergo senescence with age, and whether this is detrimental and causative of aging. Indeed, senescent cells can also be positive regulator of tissue repair (Demaria et al., 2014), and there is evidence that CR slows rates of wound healing (Hunt et al., 2012). Careful analysis on the balance between beneficial and detrimental effects of reducing senescence in various tissues upon CR will need to be addressed.

Something worth noting is that when we recorded the changes in sizes of the different organs, the alimentary tract was completely protected (and even grew a little) when compared with other organs (Mitchell et al., 2015). Clearly different organs respond very differently to the CR intervention and this may be also true for the senescence phenotype, and hence, also other features like wound healing.

## CONFLICT OF INTEREST

None Declared.
