# Supplementary material for: The effects of graded caloric restriction: XII. Comparison of mouse to human impact on cellular senescence in the colon
Source: Aging Cell. 2018 Mar 25;17(3):e12746. doi: 10.1111/acel.12746 (PMC5946078; doi:10.1111/acel.12746)
Supplement: Supplementary file 1 [file ACEL-17-e12746-s001.pdf]

SUPPLEMENTARY INFORMATION

Materials and Methods

Mice. Full experimental details are available in [1]. All procedures were reviewed and approved by University of Aberdeen ethical approval committee and carried out under a Home Office issued license compliant with the Animals (Scientific Procedures) Act 1986. The project has been registered at the open science framework (doi: 10.17605/OSF.IO/9YATH). In brief male C57/BL6 mice (Charles River, Ormiston, UK) were purchased at age 6 weeks and acclimated for 6 weeks prior to implantation of transmitters (Vital view emitters) at 12 weeks of age, allowing adequate recovery time prior to experimentation. A number of baseline measurements, including dual X-ray absorptiometry (DXA) for body composition, glucose tolerance tests (GTT) and resting metabolic rate, were carried out at 17-18 weeks old. Over the baseline period all animals were provided with *ad libitum* access to water and an open source diet (D12450B, Research Diets, NJ, USA) containing 20% protein, 70% carbohydrate and 10% fat (by energy).

Animals were randomly allocated to one of 6 groups that consisted of 4 levels of calorie restriction (10 to 40 % reduction relative to their own baseline intake) and two control groups (details below) at 20 weeks of age, approximately equivalent to early adulthood of humans. Mice continued to be fed D12450B under restriction which lasted 12 weeks. Given 24h ad libitum food access mice may become obese. Therefore an additional group where access to food was limited to the 12 hours of darkness was used as a second control and referred to as 12AL. Food intake of the 12AL and 24AL groups did not differ significantly (Mitchell et al 2015a). To minimize light phase disturbances mice were fed once per day, immediately prior to lights out and food was removed at the onset of light phase. Mice were killed on day 90 of restriction between 1400 and 1700h to minimize any circadian effects on hormone levels and body composition.

The same individual mice have been used to explore the impact of graded CR on transcriptomic responses in multiple tissues [2, 3], the liver metabolome [4], metabolic rate [5], circulating hormones [6], body composition [1], physical activity [7] and body temperature [8].

Human biopsies. Twelve individuals had been on CR for an average of 10.1 years (7-14 years). Subjects were instructed by an experienced research dietician to record all food and beverages

consumed, preparation methods, and approximate portion sizes for seven consecutive days. Food records were analyzed using the NDS-R program (version 4.03_31), which is the Nutrition Data System for research from the Nutrition Coordinating Center at the University of Minnesota. CR subjects consumed a variety of nutrient-dense unprocessed foods (i.e., vegetables, fruits, nuts, egg whites, fish, poultry, low-fat dairy products, whole grains, and beans) which supplied >100% of the recommended daily intake for all essential nutrients. Refined foods rich in empty calories and trans fatty acids were avoided. Energy intake was 30% lower in the CR group (1693±293 kcal/day) than in the age-matched Western diet (WD-o) group (n=12) (2845±509 kcal/day) (P<0.0001). The percentage of total energy intake derived from protein, carbohydrate, and fat, was 16.9%, 61%, and 27.4%, respectively, in the CR group and 14.4%, 47.4%, and 35.9% in the WD group. Moreover, a younger cohort (age, 24.3±2.0) of humans eating a typical Western diet (WD-y, n = 6) was included in this study. Energy intake of the WD-y group was 2617±712 kcal/day, and the percentage of total energy intake derived from protein, carbohydrate, and fat was 17.2%, 48.2%, and 33.4%. Height was measured without shoes to the nearest 0.1 cm. Body weight, a venous blood sample and endoscopic colonic mucosal specimens from sigmoid colon were taken after subjects had fasted for at least 12 hours. Total body fat mass and fat free mass were determined by dual-energy X-ray absorptiometry (DXA) (QDR 4500, Hologic, Waltham, MA). The human study was approved by the Human Studies Committee of Washington University School of Medicine, and all participants gave informed consent before their participation.

<u>RNA isolation and cDNA synthesis.</u> Tissues were homogenized in liquid nitrogen. For each sample, 20mg of tissue powder was used to isolate total RNA using the Isolate II RNA Mini Kit (Bioline). 250-500ng of RNA was reverse transcribed into cDNA using a kit (Applied Biosystems).

<u>Real Time-qPCR</u>. qRT-PCR reactions were performed with the LightCycler 480 Instrument II (Roche) using UPL system (Roche) with a SensiFast Probe kit (Bioline). The reactions were carried out in a total volume of 10 μl using a TaqMan assay. Tubulin was used for normalization of the CT values. List of primers/probe combination is provided below.

<u>UPL primers + probes</u>

Genes	Species	Direction	Sequence	UPL Probe
Tubulin	Mouse	Forward	ctggaacccacggtcatc	#89
		Reverse	Gtggccacgagcatagttatt	
Cdkn2a(p16)	Mouse	Forward	aatctccgcgaggaaagc	#91
		Reverse	gtctgcagcggactccat	
Cdkn1a(p21)	Mouse	Forward	aacatctcagggccgaaa	#16
		Reverse	tgcgcttggagtgatagaaa	
Il1a	Mouse	Forward	ttggttaaatgacctgcaaca	#52
		Reverse	gagcgctcacgaacagttg	
Mmp3	Mouse	Forward	ttttggccatctcttccatc	#89
		Reverse	ctcctcgtgccctcgtatag	
Mmp9	Mouse	Forward	agacgacatagacggcatcc	#83
		Reverse	tcggctgtggttcagttgt	
Cxcl1	Mouse	Forward	gactccagccacactccaac	#83
		Reverse	tgacagcgcagctcattg	
Tubulin	Human	Forward	cttcgtctccgccatcag	#40
		Reverse	cgtgttccaggcagtagagc	
Actin	Human	Forward	ccaaccgcgagaagatga	#64
		Reverse	ccagaggcgtacagggatag	
Cdkn2a(p16)	Human	Forward	gagcagcatggagcctc	#67

		Reverse	cgtaactattcggtgcgttg	
Cdkn1a(p21)	Human	Forward	tcactgtcttgtacccttgtgc	#32
		Reverse	ggcgtttggagtggtagaaa	
Il1a	Human	Forward	ggttgagtttaagccaatcca	#6
		Reverse	Tgctgacctaggcttgatga	
Mmp9	Human	Forward	gaaccaatctcaccgacagg	#53
		Reverse	gccacccgagtgtaaccata	
Cxcl1	Human	Forward	catcgaaaagatgctgaacagt	#83
		Reverse	ataagggcagggcctcct	

Statistical analysis. One-way analysis of variance (ANOVA) was used to compare group variables, followed by Tukey post-hoc testing when indicated. One-way ANOVA with Games-Howell was performed for distributions where equal variances could not be assumed. Statistical significance was set at $P < 0.05$ for all tests. All data were analyzed by using SPSS software, version 25.0 (SPSS Inc, Chicago). Data are expressed as mean ± SD in Figure 2A or mean ± SEM otherwise. A difference with P-values < 0.05 were considered statistically significant.

1. Mitchell, S.E., Tang, Z., Kerbois, C., Delville, C., Konstantopedos, P., Bruel, A., Derous, D., Green, C., Aspden, R.M., Goodyear, S.R., et al. (2015). The effects of graded levels of calorie restriction: I. impact of short term calorie and protein restriction on body composition in the C57BL/6 mouse. Oncotarget *6*, 15902-15930.
2. Derous, D., Mitchell, S.E., Green, C.L., Wang, Y., Han, J.D.J., Chen, L., Promislow, D.E.L., Lusseau, D., Douglas, A., and Speakman, J.R. (2017). The Effects of Graded Levels of Calorie Restriction: X. Transcriptomic Responses of Epididymal Adipose Tissue. J Gerontol A Biol Sci Med Sci.
3. Derous, D., Mitchell, S.E., Green, C.L., Chen, L., Han, J.D., Wang, Y., Promislow, D.E., Lusseau, D., Speakman, J.R., and Douglas, A. (2016). The effects of graded levels of calorie restriction: VI. Impact of short-term graded calorie restriction on transcriptomic responses of the hypothalamic hunger and circadian signaling pathways. Aging (Albany NY) *8*, 642-663.

4. Green, C.L., Mitchell, S.E., Derous, D., Wang, Y., Chen, L., Han, J.J., Promislow, D.E.L., Lusseau, D., Douglas, A., and Speakman, J.R. (2017). The effects of graded levels of calorie restriction: IX. Global metabolomic screen reveals modulation of carnitines, sphingolipids and bile acids in the liver of C57BL/6 mice. Aging Cell *16*, 529-540.
5. Mitchell, S.E., Tang, Z., Kerbois, C., Delville, C., Derous, D., Green, C.L., Wang, Y., Han, J.J., Chen, L., Douglas, A., et al. (2017). The effects of graded levels of calorie restriction: VIII. Impact of short term calorie and protein restriction on basal metabolic rate in the C57BL/6 mouse. Oncotarget *8*, 17453-17474.
6. Mitchell, S.E., Delville, C., Konstantopedos, P., Hurst, J., Derous, D., Green, C., Chen, L., Han, J.J., Wang, Y., Promislow, D.E., et al. (2015). The effects of graded levels of calorie restriction: II. Impact of short term calorie and protein restriction on circulating hormone levels, glucose homeostasis and oxidative stress in male C57BL/6 mice. Oncotarget *6*, 23213-23237.
7. Mitchell, S.E., Delville, C., Konstantopedos, P., Derous, D., Green, C.L., Wang, Y., Han, J.D., Promislow, D.E., Douglas, A., Chen, L., et al. (2016). The effects of graded levels of calorie restriction: V. Impact of short term calorie and protein restriction on physical activity in the C57BL/6 mouse. Oncotarget *7*, 19147-19170.
8. Mitchell, S.E., Delville, C., Konstantopedos, P., Derous, D., Green, C.L., Chen, L., Han, J.D., Wang, Y., Promislow, D.E., Douglas, A., et al. (2015). The effects of graded levels of calorie restriction: III. Impact of short term calorie and protein restriction on mean daily body temperature and torpor use in the C57BL/6 mouse. Oncotarget *6*, 18314-18337.
